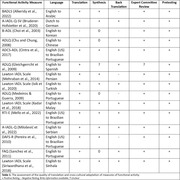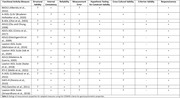# A Systematic Review on the Process of Translation and Cross‐Cultural Adaptation of Functional Activity Tests for Dementia

**DOI:** 10.1002/alz.095037

**Published:** 2025-01-09

**Authors:** Freddie O'Donald, Clara Calia

**Affiliations:** ^1^ University of Edinburgh, Edinburgh United Kingdom; ^2^ NHS Tayside, Dundee United Kingdom

## Abstract

**Background:**

This systematic review aims to investigate the methodologies employed in the translation and cross‐cultural adaptation of functional activity measures for dementia on a global scale. The Consensus‐based Standards for the Selection of Health Measurement Instruments (COSMIN) initiative is used as a framework, offering established criteria for assessing psychometric properties across diverse domains.

**Method:**

A systematic search of five electronic databases (CINAHL Plus, EMBASE, PubMed, MEDLINE, PsycINFO) was conducted from inception until September 2023. Quality assessment criteria were then utilised to evaluate the process of cross‐cultural adaptation and psychometric properties of identified functional activity measures.

**Result:**

Fifteen studies relating to adapted functional activity measures in eleven languages were identified. It was found that less than half of these studies fully adhered to established guidelines for the translation and cross‐cultural adaptation of instruments. Regarding psychometric properties, while the internal consistency and reliability of included measures were generally strong, there was variability in evaluating other psychometric properties, notably structural validity, measurement error, and cross‐cultural validity.

**Conclusion:**

This review underscores the need for researchers and clinicians to follow standardised guidelines for translating and cross‐culturally adapting functional activity measures for dementia and ensuring the comprehensive evaluation of psychometric properties in cross‐cultural settings. Researchers and clinicians should consider whether the psychometric properties and characteristics of an adapted functional activity measure are suitable for use in their population of interest.